# Supplementary figures and images for: Exploring the In Vitro and In Vivo Therapeutic Potential of BRAF and MEK Inhibitor Combination in NRAS-Mutated Melanoma
Source: Cancers (Basel). 2023 Nov 22;15(23):5521. doi: 10.3390/cancers15235521 (PMC10705743; doi:10.3390/cancers15235521)

# Uncropped Western Blots Figure 2D

Actin

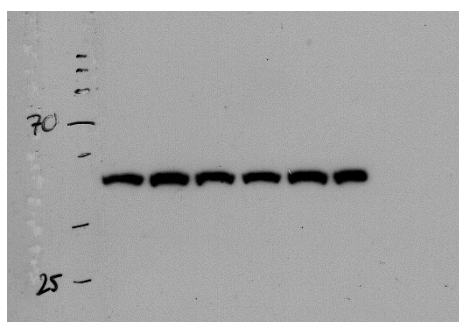

AKT

pERK

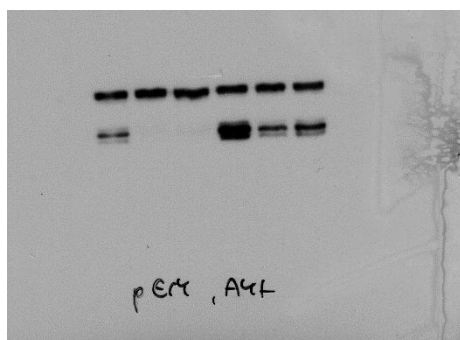

pAKT

ERK

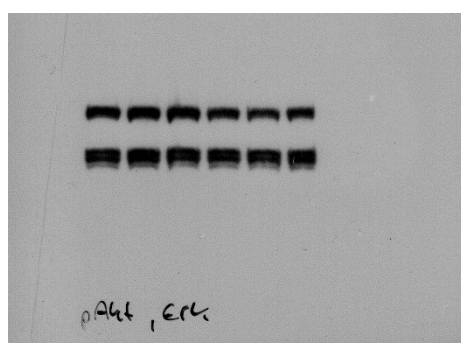

ATF4

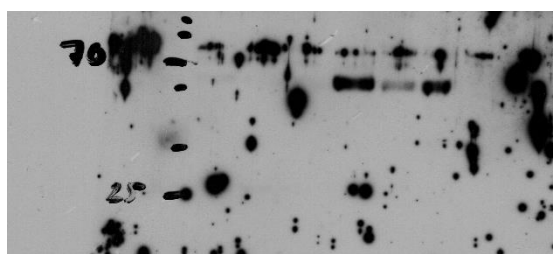

Supplement: Supplementary file 1 [file cancers-15-05521-s001.zip › cancers-2653537-supplementary.pdf]
